# Mjölnir: a miniature triaxial rock deformation apparatus for 4D synchrotron X-ray microtomography

**DOI:** 10.1107/S160057752001173X

**Published:** 2020-10-16

**Authors:** Ian Butler, Florian Fusseis, Alexis Cartwright-Taylor, Michael Flynn

**Affiliations:** a School of Geosciences, University of Edinburgh, James Hutton Road, The King’s Buildings, Edinburgh EH9 3FE, United Kingdom

**Keywords:** synchrotron X-ray microtomography, experimental geoscience, rock deformation

## Abstract

An X-ray transparent experimental triaxial rock deformation apparatus, here named ‘Mjölnir’, enables investigations of brittle-style rock deformation and failure, as well as coupled thermal, chemical and mechanical processes relevant to a range of Earth subsurface environments. Designed to operate with cylindrical samples up to 3.2 mm outside-diameter and up to 10 mm length, Mjölnir can attain up to 50 MPa confining pressure and in excess of 600 MPa axial load. The addition of heaters extends the experimental range to temperatures up to 140°C. Deployment of Mjolnir on synchrotron beamlines indicates that full 3D datasets may be acquired in a few seconds to a few minutes, meaning full 4D investigations of deformation processes can be undertaken. Mjölnir is constructed from readily available materials and components and complete technical drawings are included in the supporting information.

## Abstract 2

The design, construction and assembly details for an X-ray transparent triaxial deformation cell for 4D investigations of rock deformation and coupled chemical–mechanical processes in the geosciences are presented. The portability and flexibility of the cell are demonstrated by deployment on four different synchrotron beamlines.

## 1. Introduction   

The accumulation of damage in rocks and their ultimate failure under deviatoric stress is a significant process in a range of natural and engineered settings within the Earth’s sub­surface. The mechanical behaviour of rocks is also influenced by a range of chemical processes including dissolution, mineral dehydration and volume changes during metamorphic reactions. Our understanding of such processes in the Earth’s subsurface has been greatly advanced by a range of physical and chemical experimental approaches used to replicate processes at elevated pressure and temperature. Conventional laboratory experiments, typically using cylindrical cores of rock 25–100 mm in diameter, demand that the experimental apparatus be constructed of robust materials possessing both high tensile strength and stiffness. These materials, and the need to construct apparatus that must themselves be stiff (*i.e.* resistant to deformation by the applied stresses), have precluded the application of high-resolution X-ray microtomography techniques to directly observe the dynamic processes during rock deformation and reaction processes at elevated pressure and temperature. As a consequence, many advances in our understanding of deformation processes, failure mechanisms and the role of fluid rock reaction have been achieved either indirectly by determination of bulk properties, by means of comparatively low-resolution methods such as locating acoustic emissions from microfracturing events (*e.g.* Lockner *et al.*, 1991 ▸; Aben *et al.*, 2019 ▸) or by post-mortem evaluations of samples.

Recently the opportunity to use X-ray microtomography to image deformation processes *in situ* has been facilitated by the development of miniaturized rock deformation cells that operate on laboratory micro-computed tomography (µCT) instruments or at synchrotron beamlines (*e.g.* Viggiani *et al.*, 2004 ▸; Lenoir *et al.*, 2007 ▸; Tisato *et al.*, 2014 ▸; Renard *et al.*, 2016 ▸; Glatz *et al.*, 2018 ▸; Voltolini *et al.*, 2019 ▸). In this contribution, we outline the design, construction and deployment of a miniature rock-deformation press suitable for synchrotron X-ray microtomography studies. This new press, which we have named ‘Mjölnir’ (in Norse mythology Mjölnir is the hammer wielded by Thor, the god of thunder), is a partner to the elevated pressure (P) and temperature (T) fluid rock reaction apparatus described in the work by Fusseis *et al.* (2014 ▸). We provide CAD-drawings (see supporting information) and details of the design, construction and operation of the cell so that researchers can replicate or further develop Mjölnir for their own scientific applications. Mjölnir differs from previously published cell designs primarily through simplicity of design and construction. Although unable to approach the upper temperature limits of the cells presented in the work by Voltolini *et al.* (2019 ▸) and Glatz *et al.* (2018 ▸), Mjölnir exceeds their capability in confining pressure and axial load. The HADES cell presented in the work by Renard *et al.* (2016 ▸) can operate under higher P and T conditions than Mjölnir in its present format, yet has the limitation that its use is currently tied to a single, high-energy synchrotron beamline. We demonstrate that Mjölnir is flexible in deployment across several synchrotron sources and can operate on relatively low-energy polychromatic and monochromatic tomographic imaging beamlines.

Initially designed to provide high-resolution 4D imaging of the accumulation and localization of damage in the lead up to brittle failure of rocks at elevated pressure, we have been able to extend the operation of the press to temperatures up to 140°C to investigate coupled chemical and mechanical processes (*i.e.* processes which exert positive or negative feedbacks upon each other, and so influence the progressive evolution of the structure, properties or behaviour of a material). Mjölnir is built from commercially available materials and components, has a modest cost of construction and can be deployed on a range of synchrotron beamlines.

## 2. Design and construction   

Mjölnir’s configuration is consistent with the geometry of a conventional Hoek cell commonly used in triaxial rock deformation experiments (Hoek & Franklin, 1968 ▸). As such, the principal orthogonal stresses σ_1_, σ_2_ and σ_3_ are configured such that the total axial stress σ_1_ = (σ + *p*) > (σ_2_ = σ_3_ = *p*) as the sample is deformed, where σ is the differential stress and *p* is the radial confining pressure.

**Figure 1:**
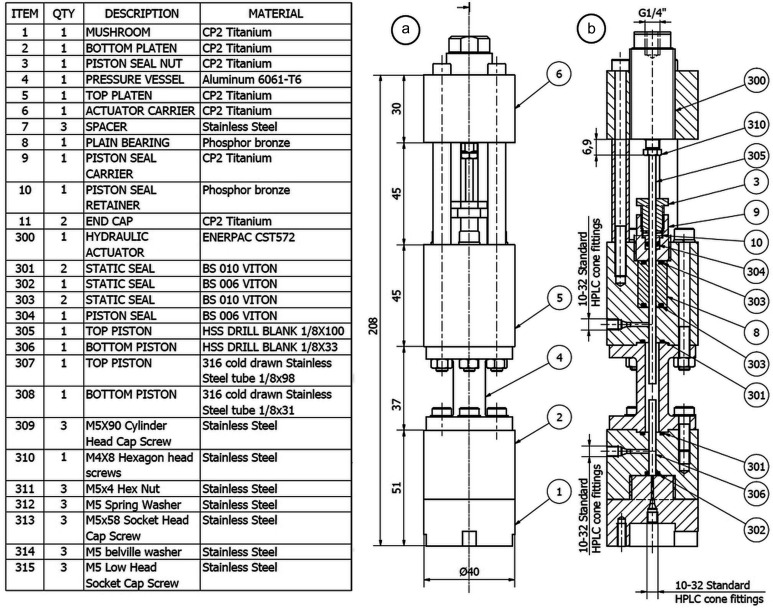
Bill of materials, (*a*) elevation and (*b*) sectional drawing of Mjölnir illustrating the overall design, components and construction of the cell. CAD-drawings for each component are provided in folder S1 of the supporting information.

To attain a small, light configuration for fast synchrotron tomography, Mjölnir has been designed to operate with 3–3.2 mm-diameter cylindrical rock samples up to 10 mm in length. The fully assembled cell weighs just over 1 kg and stands 225 mm tall. It is constructed of CP2 grade titanium, 316 grade stainless steel, phosphor bronze alloy, 6061 aluminium alloy (T6 temper) and 12.9 grade high tensile steel. All o-ring seals are Viton or PTFE. The assembled components and materials utilized for each component are specified in the bill of material (Fig. 1 ▸) and CAD-drawings are provided in the supporting information.

The cylindrical sample is held between two pistons and sleeved using a flexible silicone (Tygon) sleeve. The pistons can be made from either high-speed steel (HSS) drill blanks or cold-drawn 316 stainless steel high-pressure tubing. The axial load delivered to the sample via the top piston is generated by means of an Enerpac CST572 single-acting hydraulic actuator with a maximum stroke of 7 mm. The actuator exerts a 4.4 kN force when pressurized to 35 MPa (equal to the 350 bar actuator operating limit, and equivalent to >600 MPa on a 3 mm sample). Fluid connections made directly to Mjölnir’s body are with 10/32 high-performance liquid chromatography (HPLC) fittings. Fluid connections to the Enerpac actuator use Swagelok tube unions.

A critical element of the design is the pressure cell, which must offer minimal X-ray attenuation, yet must be stiff enough to resist the stresses imposed by the pressurized confining fluid and axial load. We selected aluminium alloy 6061 for the pressure vessel which has a yield strength in T6 temper of 276 MPa at 24°C (ASM Handbook, 1990 ▸). The material is corrosion resistant and contains no alloying components with X-ray attenuation exceeding that of Al at concentrations of >1 wt%. Mjölnir’s pressure vessel is designed with a 4 mm-thick wall (inner diameter 6 mm, outer diameter 14 mm). Guideline calculations of axial stress at full actuator load (4.4 kN) and thick-walled cylinder hoop and radial stresses at 50 MPa confining pressure indicate that the axial stress (35 MPa) on the wall and combined hoop and radial stresses on the inner surface of the cell (122 MPa) are, in total, substantially less than the yield strength of the 6061 aluminium alloy, even at 150°C.

Pistons are either 1/8-inch HSS aircraft extension drill blanks for use with non-porous materials, or cold-drawn 1/8-inch 316 stainless steel high-pressure tubing for those instances when pore fluid pressure and/or thermocouple access to the sample are desired. In both cases, the material is stiff enough to resist deformation. Commonly available 316 stainless steel tubing supplied in the annealed state was found to be too soft for use as piston material, even at low axial loads. The addition of CP2 grade Ti tips to the pistons (attached using 3M Scotch-Weld DP760 high-temperature structural ep­oxy) significantly improves X-ray imaging of samples close to the piston ends as this reduces artefacts resulting from the high absorption contrast between a rock sample and the steel piston. The Ti tips also reduce the bore dimension for fluid access to the sample to 0.4 mm. The dynamic seal on the top piston (component 304, Fig. 1 ▸) is made with a single BS006 o-ring without a back-up ring. Although a dynamic o-ring seal would conventionally require a back-up ring to avoid extrusion at the pressures applied in Mjölnir, the piston displacement rate and length of travel is sufficiently limited that the seal operates without leaking provided that the phosphor bronze seal retainer (component 10, Fig. 1 ▸) is manufactured to be a close fit to the o-ring groove in the seal housing.

A phosphor bronze plain bearing situated in the upper piston retainer acts as a linear guide to ensure that the piston, driven by the hydraulic actuator, runs accurately. The upper piston retainer of the cell is the component which requires the most time-consuming machining, but the inclusion of a replaceable plain bearing adds flexibility to accommodate small variations in piston diameter.

### 2.1. Modification of the basic design   

Mjölnir’s components are modular, and can be modified, exchanged and adapted to meet the requirements of individual experiments. The operational limits of any implementation of Mjölnir can be estimated from engineering calculations but must be established, in practice, by testing the cell to beyond the expected operating parameters of confining pressure, axial load and temperature. Each modification of the cell design, especially where structural components are altered, or operating conditions (pressure and/or temperature) are increased, requires reassessment by both calculation and testing under safe conditions. The cell is designed for use with liquid-pressure media only. Depending on the usage history, load-bearing components must be replaced at regular intervals.

**Figure 2:**
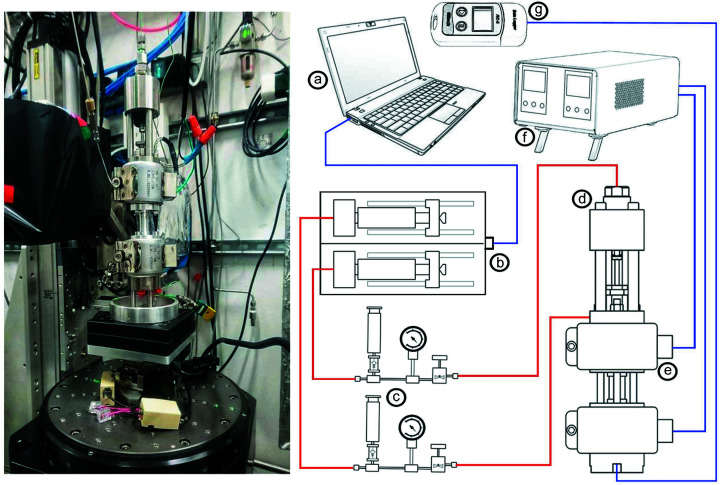
(Left-hand side) Mjölnir in position and set up for operation on the rotary table on beamline 2-BM of the Advanced Photon Source. (Right-hand side) Cartoon schematic of the layout and connection of control/power/signal (blue) and fluid (red) connections for typical operation. (*a*) Laptop PC with Qmix Elements software controls (*b*) two Cetoni neMESYS high-pressure syringe pumps which are connected via (*c*) fluid manifolds to the (*d*) hydraulic actuator and confining pressure vessel of Mjölnir. (*e*) Band heaters fitted to Mjölnir are controlled via (*f*) Watlow temperature controllers, and the sample temperature is monitored via a data-logger or temperature display connected to a thermocouple in contact with the base of the sample.

The design presented above can be modified in order to enable experiments to be conducted at elevated temperature in combination with elevated confining pressure and axial load. This enables improved replication of conditions found in the Earth’s subsurface environment, and which may be relevant to fluid rock reaction rates. The modest size of the cell, and the requirement to maintain the X-ray beam-path free of extraneous objects, means that the simplest way to supply heat to the sample utilizes band heaters (Fig. 2 ▸) employed in the same manner as in the work by Fusseis *et al.* (2014 ▸). Two band heaters, each of 270 W capacity, surrounding the top and bottom platens of Mjölnir enable the sample to be heated to 130°C, with the band heaters maintained at 140°C. Control of the band heaters uses Watlow EZ-ZONE controllers driving Watlow DIN-A-MITE solid-state power controllers. For each band heater, a type-K control thermocouple is clamped between the band heater and the body of Mjölnir to provide temperature feedback to the controller for that heater. The sample temperature can be monitored by a stainless steel sheathed 0.75 mm-diameter type-K thermocouple inserted through the HPLC port at the base of Mjölnir. The thermocouple tip should be positioned as close to the sample as possible. For elevated-temperature applications (especially if water is used as a pressure medium) it may prove beneficial to replace the phosphor bronze plain bearing with one made from the same material as the top platen of Mjölnir (component 5 in Fig. 1 ▸) or to adapt the design to remove the plain bearing altogether.

Higher operating temperatures may be attainable with substitution of suitable materials with better elevated-temperature performance than either CP2 grade Ti or 6061 Al alloy. The upper temperature limit for safe operation of Mjölnir, as presented herein, is limited because 6061-T6 alloy retains >90% of its room-temperature yield strength up to 100°C, but begins to decline in its mechanical properties in the region between 100°C and 150°C and beyond. At 150°C its yield strength is 77% of its room-temperature value, and falls to 37% of that value at 200°C (ASM Handbook, 1990 ▸). This trend is common for readily available Al alloys. All commercial pressure fittings and tubing used for each experiment are rated for pressures consistent with or beyond the operational limitations of the cell presented here.

## 3. Operation of the cell and data acquisition   

### 3.1. Sample preparation   

Samples must be prepared as cylinders of constant radial dimension with flat ends perpendicular to the long axis. Cylindrical samples of 3 mm diameter and up to 10 mm long are best obtained by means of coring with a thin-walled diamond core drill in a water-flushed chuck attached to a pillar drill. Some soft materials such as limestone and alabaster have proven difficult to core at this size by this method, and in such cases larger diameter cores can be turned down to the desired diameter by means of a lathe and carbide-tipped tools. In all cases the sample ends should be ground flat and perpendicular to the long axis by mounting the core in a collet on a lathe and grinding to a flat surface using a diamond-impregnated abrasive disk mounted on the lathe tool-post.

### 3.2. Cell assembly   

Assembly of Mjölnir is straightforward. Full guided assembly instructions are included in the supporting information and a cartoon of the complete experimental assembly is illustrated in Fig. 2 ▸. The sample is placed between the upper and lower pistons and sheathed with a flexible silicone jacket (commercially produced 1/8-inch bore, 1/32-inch wall tubing) which is held in place over the piston ends using twisted wire loops (*cf*. Fusseis *et al.*, 2014 ▸). We found that silicone tubing is incompatible with many silicone oils due to polymer swelling, and in particular at elevated temperature. We used deionized water as a compatible pressure medium throughout our experiments.

Adapter plates to install the cell onto a rotary table need to be created to suit the specific synchrotron beamline on which Mjölnir is to be employed. Once in place on the rotary table of the beamline, all fluid connections can be made and band heaters attached (if required). All pressurized fluid connections from our pumps to Mjölnir are made with flexible PEEK (polyether ether ketone) HPLC tubing. It is the pressure rating of flexible PEEK tubing (no greater than 50 MPa for 1/16-inch tubing with a bore of 0.01-inch) that presents the upper operational limit with fluid pressure delivered to the cell on the rotary table via flexible tubes.

The PEEK tubing is attached via 1/16-inch Swagelok unions to 1/16-inch stainless steel tubing which is connected to Mjölnir itself via stainless steel HPLC fittings (see Fig. 2 ▸). The 1/16-inch stainless steel tube enables a tighter bend radius than can be achieved using PEEK tubing and offers a rigid and reliable connection to the cell body. Our experience has been that with suitable strain relief on fluid connections and through the avoidance of tight-bend radii, PEEK tubing can be employed for extended periods of operation involving multiple (hundreds to thousands) rotations without damage during tomographic data acquisition.

In our experiments, both confining pressure and actuator fluid lines were brought down to Mjölnir via a gantry mounted sufficiently high above the rotary stage to allow for free rotation of Mjölnir and to keep the X-ray beam path clear. Where band heaters were used, the power and thermocouple wires were also suspended from the same gantry. For the pore fluid pressure or thermocouple connections attached to the cell below the sample, along with a band heater, the fluid, signal and power lines were brought in to Mjölnir from the side of the stage. Care was taken in establishing these connections to ensure that power or fluid lines could neither snag on objects around the rotary stage nor flex upwards to interfere with the X-ray beam path.

### 3.3. Cell operation   

In our setup, fluid pressure is generated by means of Cetoni neMESYS high-pressure syringe pumps controlled remotely from a laptop using *QMix* elements software, which offers stable PID control of pump flow rate and pressure. The internal pressure sensors of the pumps enable pressure control within ±0.03 MPa, although more precise measurement and control can be attained via the addition of external, high-accuracy pressure transducers. Each pump is attached to Mjölnir via a Swagelok manifold which facilitates refilling of the pump syringes when required without the need to disconnect the pumps.

Upon loading the cell, initially the target confining pressure is established in the pressure vessel of Mjölnir and maintained under active PID control. The way the axial load is applied depends on the kind of test to be conducted. To achieve a specified strain rate the axial load is applied by pumping fluid into the hydraulic actuator at a calculated rate. Both continuous pumping and stepwise pumping (during which the pump advance was halted to enable data acquisition) are possible, and using the pumps and actuator specified, continuous strain rates as low as 10^−6^ s^−1^ can be achieved. In early configurations of Mjölnir, strain was determined post experiment by measuring the distance between pistons on µCT images. More recently we have installed a linear variable displacement transducer (LVDT) clamped to the actuator carrier which measures the displacement of a tell-tale clamped onto the top piston. Installation of an LVDT means that Mjölnir can be employed for both stress- and strain-controlled loading. Creep experiments which maintain constant differential stress states (σ_1_ > σ_2_ = σ_3_) can also be readily established.

### 3.4. Example deployments   

**Figure 3:**
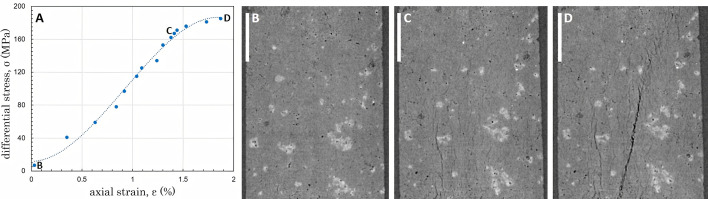
(A) Stress–strain curve and (B)–(D) example tomographic data acquired during a triaxial deformation experiment using thermally damaged Ailsa Craig microgranite (scale bar = 850 µm) at the PSICHÉ beamline, Synchrotron Soleil. Strain data in (A) were determined directly from the reconstructed images acquired at static stress points during loading. The tomographic slices shown are cut parallel to the principle stress axis (σ_1_) and show the progressive development of microfractures from (B) 7 MPa differential stress, (C) through 162 MPa differential stress, (D) to 185 MPa peak differential stress just prior to shear failure.

Mjölnir was first deployed on the PSICHÉ beamline of Synchrotron Soleil, near Paris, France, and has subsequently been deployed on beamline 2-BM of the Advanced Photon Source (APS), Argonne National Laboratory, Chicago, USA, twice at the TOMCAT beamline at the Swiss Light Source (SLS), Paul Scherrer Institute, and at the I12 Joint Engineering, Environmental and Processing (JEEP) beamline of the Diamond Light Source (DLS), UK. Example data acquired at some of these beamlines are presented in Figs. 3 ▸, 4 ▸ and 5 ▸. Experimental applications thus far have included observation of the mechanical failure and prelude to failure of micritic limestone and of fresh and heat-treated Ailsa Craig microgranite samples (Cartwright–Taylor *et al.*, 2020 ▸) as well as investigation of coupled thermal–chemical–mechanical processes during the dehydration of gypsum to bassanite (Marti *et al.*, 2020 ▸).

Ambient-temperature rock deformation experiments were executed on the PSICHÉ beamline (Cartwright–Taylor *et al.*, 2020 ▸). For these experiments data were acquired using pink beam radiation by filtration of the white beam (15–100 keV) by means of 1 mm aluminium and 0.5 mm tungsten filters. During each scan, 1200 projections were acquired over 180°, with an exposure time per projection of 15–19 ms, depending on the progressive darkening of the objective lens. Between each scan the cell was translated vertically to produce three to four overlapping scans which were stitched together to cover the entire sample volume to yield 3D volumes of 1700 × 1700 × 4102 equidimensional voxels of 2.7 µm edge length. During these experiments the sample was loaded in a staircase ramp, with the sample load held static during each group of four scans. The maximum axial load the sample experienced at 30 MPa confining pressure was 235 MPa (205 MPa differential stress). Axial strain was determined by measuring the distance between the piston surfaces in the reconstructed tomographic volumes. Example data illustrating the experimental stress–strain curve and features pre- and post-failure are shown in Fig. 3 ▸.

Experiments at beamline 2-BM of the APS, at the TOMCAT beamline of the SLS and at the JEEP beamline of DLS focused on coupled chemical and mechanical processes during mineral-dehydration reactions. For these the dehydration of gypsum (Volterra alabaster) was used as an analogue for the structural and mechanical changes associated with dehydration reactions at high pressure and temperature.

**Figure 4:**
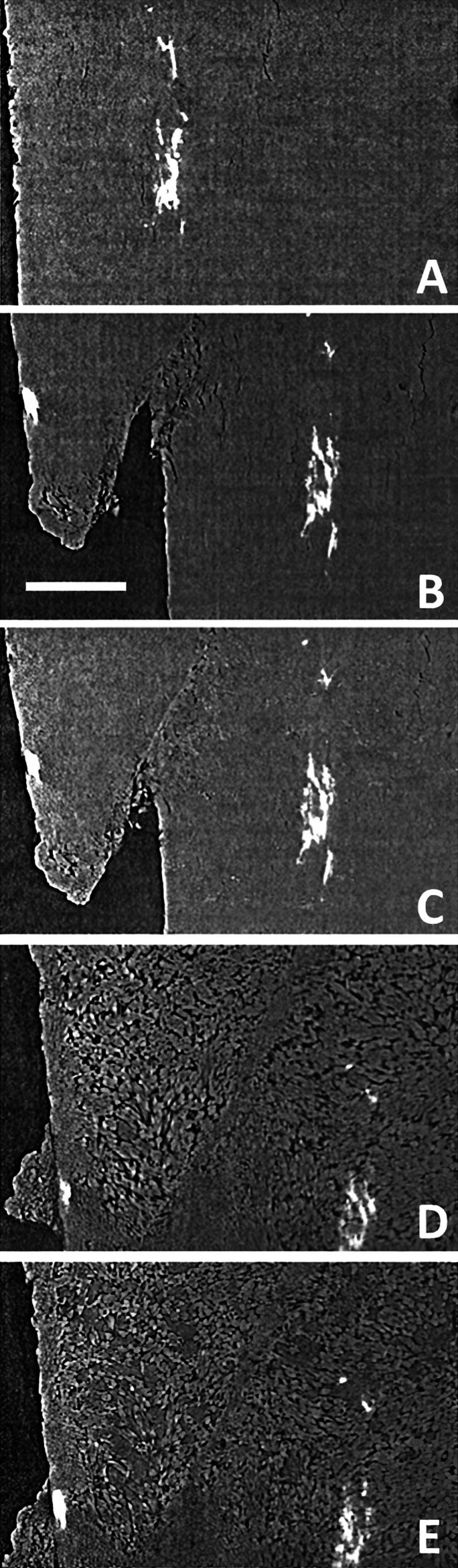
Example tomographic data acquired from beamline 2-BM at the Advanced Photon Source. A sequence of images representing vertical slices (parallel to σ_1_) from reconstructed volumes of Volterra alabaster is shown in (A)–(E) (scale bar = 250 µm). Initially, pristine alabaster (A) begins to dehydrate from gypsum to bassanite [speckled area in (B)] which weakens the sample, promoting shear failure [(B) and (C)]. Further dehydration [(D) and (E)] both creates additional porosity and, apparently, leads to ‘healing’ of the plane of shear failure (E).

At the APS, data were acquired using pink beam radiation produced by filtration of the white beam by 1 mm carbon, 2 mm silica and 1 mm glass filters to yield a photon flux with a peak energy of 65 keV. Data were acquired at a 30° s^−1^ rotation velocity, with 1500 exposures of 4 ms collected over 180°, each tomographic scan lasting 6 s. Data were acquired continuously, with 180 s between scans. Data volumes of 2160 × 2160 × 2560 voxels were acquired with a voxel edge dimension of 2.2 µm. Experiments were conducted as creep experiments under constant conditions with temperature of 115°C (±1°C), confining pressure of 15 MPa and differential stress of 38 MPa. Example data illustrating the coupling of reaction and mechanical failure are shown in Fig. 4 ▸.

**Figure 5:**
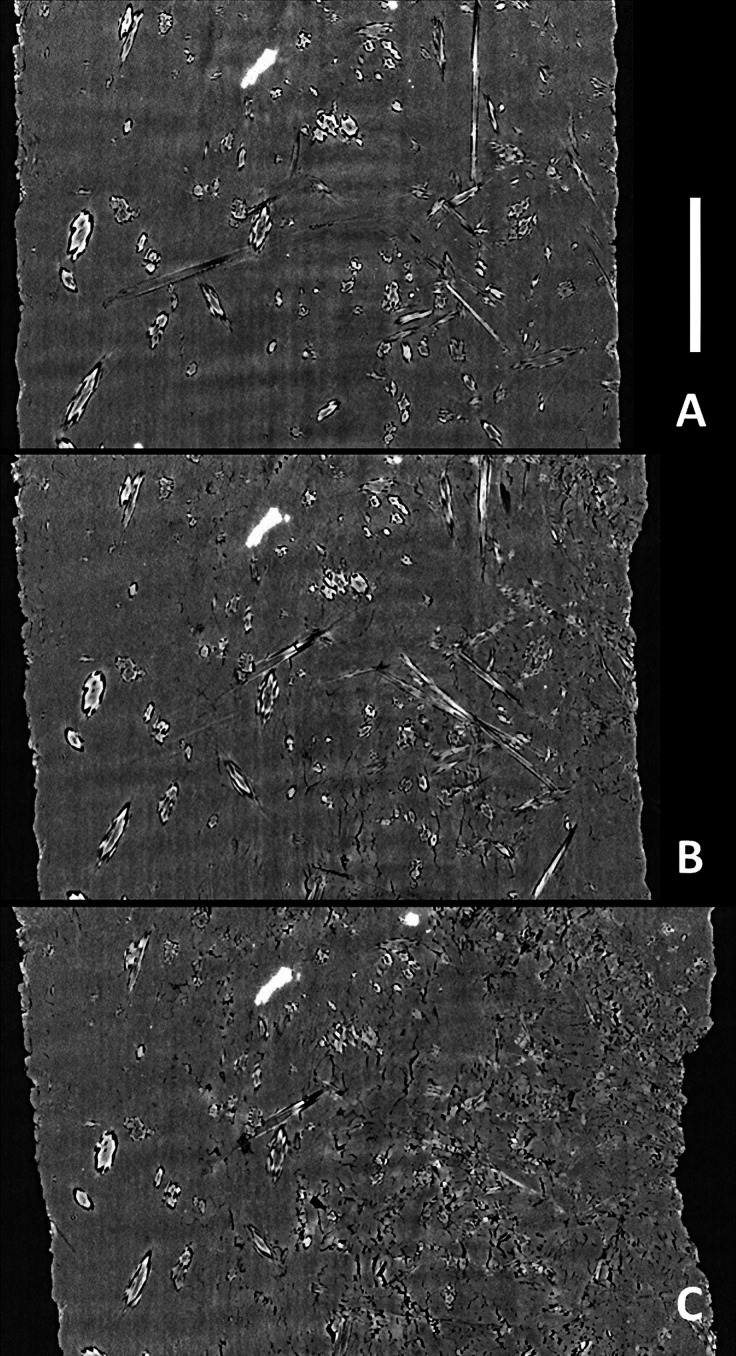
Data from gypsum dehydration experiments carried out at the TOMCAT bealine of the Swiss Light Source. Three images [(A)–(C) scale bar = 575 µm] representing vertical slices (parallel to σ_1_) from reconstructed volumes of Volterra alabaster show progressive dehydration of gypsum (mid grey) to bassanite (light grey) which grows as acicular crystals (A) surrounded by a dark moat which represents fluid-filled porosity. As bassanite crystals and their associated porosity impinge upon each other (B) the sample becomes mechanically weaker and begins to deform and collapse [(B) and (C)].

At TOMCAT, data were acquired with a pink beam (peak energy 40 keV) produced by filtration of the white beam which was filtered by 20 mm of carbon (Sigradur glassy carbon) plus 75 µm of molybdenum foil. For each scan, 2000 projections were acquired with a 1 ms exposure over 180° with a total duration of 2 s. Data volumes of 2016 × 2016 × 2016 voxels were acquired with a voxel edge dimension of 2.75 µm. Experiments were conducted as creep experiments with conditions constant for individual experiments. The range of conditions across experiments was temperature 96–110°C, confining pressure 15 MPa and differential stress 8 MPa. Data were acquired at intervals from 0.5 s to 10 min over experimental durations of 97 min to 36 h. Example data from these experiments illustrating a progressive mineral dehydration reaction are shown in Fig. 5 ▸.

At JEEP, data were acquired using a monochromatic beam of 53 keV energy, with 1500 projections of 30 ms exposure time over 180°, giving scan durations of 45 s. Data volumes of 2160 × 2160 × 2560 were obtained with voxel edge dimensions of 3.25 µm. The scan frequency was 1–5 min, subject to the experiment. Experimental conditions were constant for individual experiments and were in the range *T* = 96–115°C, confining pressure 15–20 MPa and differential stress 8–23 MPa.

The beamline and experimental conditions listed above, and the example data presented in Figs. 3 ▸, 4 ▸ and 5 ▸, confirm that Mjölnir can be used to produce high-quality, highly spatially and temporally resolved imaging of dynamic processes associated with rock deformation and deformation coupled with fluid rock reaction under realistic Earth subsurface conditions.

## 4. Conclusions   

We present the technical drawings and operational details for a simple experimental cell that enables microtomographic visualization of the damage and deformation of rocks during triaxial tests to failure at elevated pressures. The addition of heaters to the cell further facilitates fluid rock reactions to be explored under conditions of increasing or constant differential stress. It is designed for use at synchrotron light sources and optimized for portability, and has been demonstrated to enable data acquisition in the seconds to minutes range for scans of selected sub-volumes of a sample or multiple stitched volumes that cover the complete core. The modularity of the design means that the components can be replaced to enable higher pressure or temperature operation, or to enable greater X-ray transparency. The small sample size is favourable for high-resolution imaging, enabling close inspection of physical and chemical effects at the grain scale in many rocks. The 4D experiments enabled by the cell will contribute to our understanding of the fundamental physical processes associated with the accumulation of damage and its localization in geological materials as well as addressing processes relevant to aquifers, hydro­carbon reservoirs, geothermal reservoirs, CO_2_ sequestration sites and high-level radioactive waste repositories.

## Supplementary Material

Technical drawings and inventor files. DOI: 10.1107/S160057752001173X/pp5161sup1.zip

Scheme for the assembly of Mjolnir. DOI: 10.1107/S160057752001173X/pp5161sup2.pdf
