# Supplementary material for: Mjölnir: a miniature triaxial rock deformation apparatus for 4D synchrotron X-ray microtomography
Source: J Synchrotron Radiat. 2020 Oct 16;27(Pt 6):1681–7. doi: 10.1107/S160057752001173X (PMC7642963; doi:10.1107/S160057752001173X)
Supplement: Supplementary file 1 [file s-27-01681-sup1.zip › SUPPLEMENTARY INFORMATION S1 MJOLNIR CAD DRAWINGS.pdf]

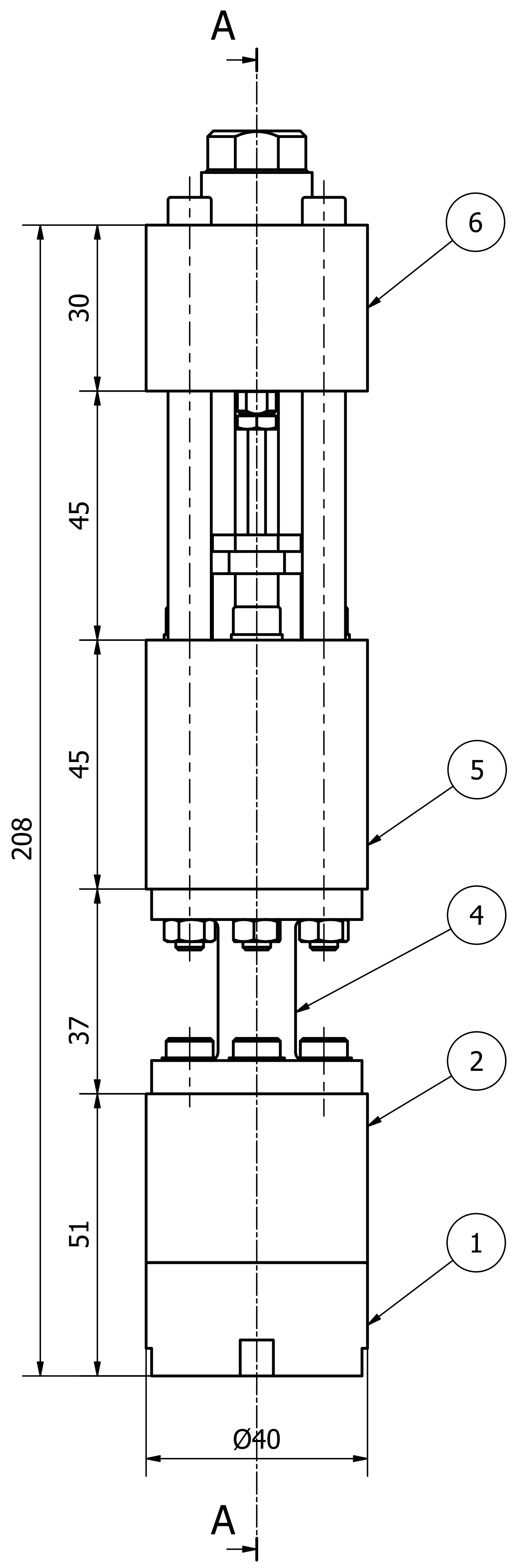

ACTUATOR IN

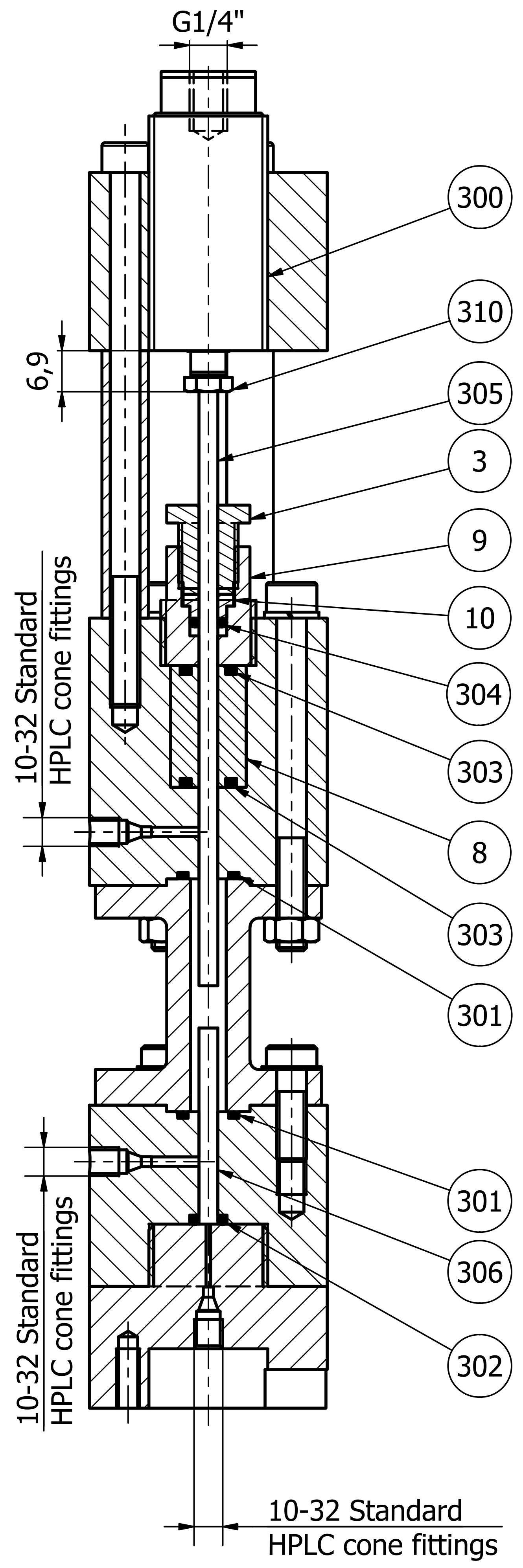

ACTUATOR OUT

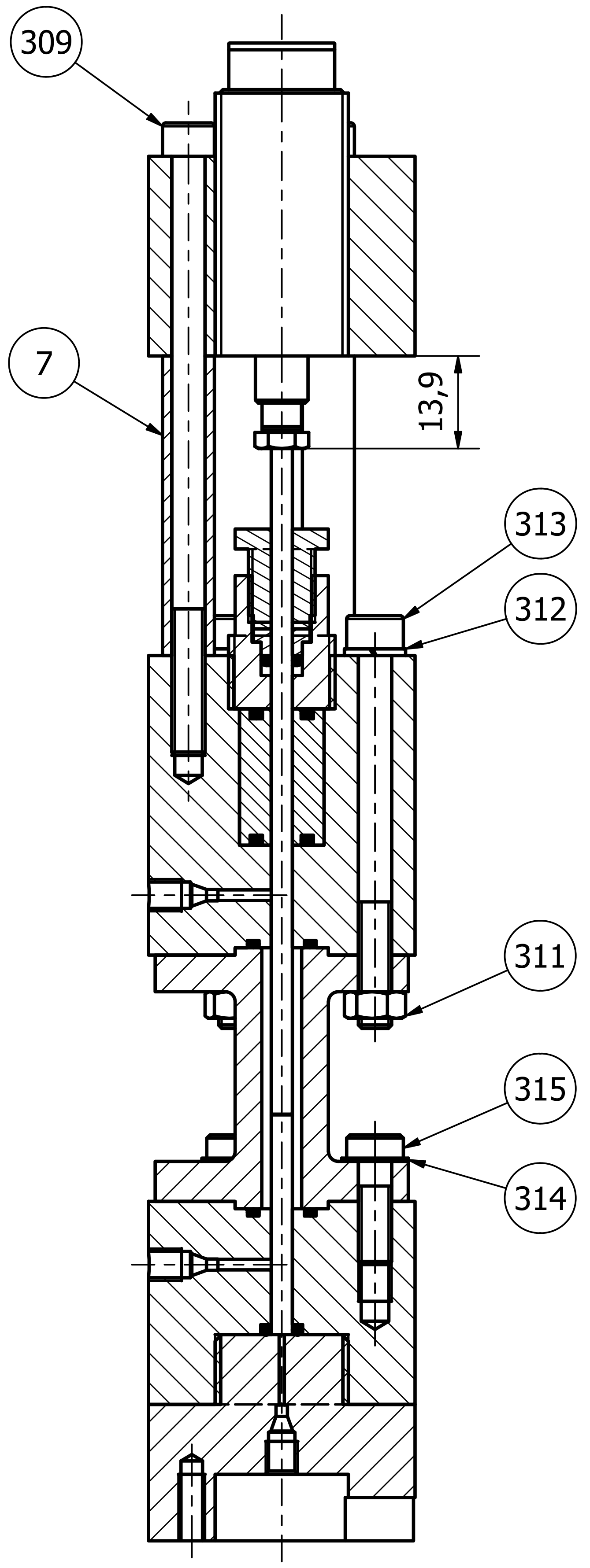

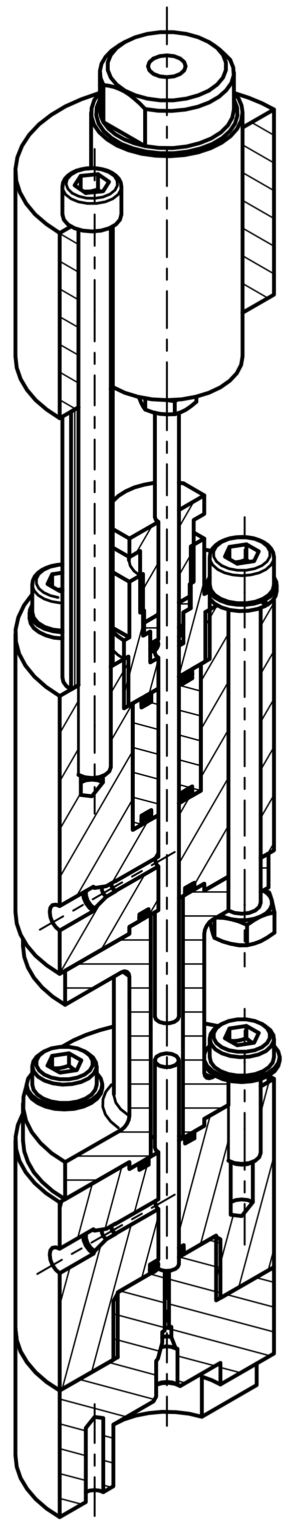

BILL OF MATERIAL

ITEM	QTY	DESCRIPTION	MATERIAL
1	1	MUSHROOM	CP2 Titanium
2	1	BOTTOM PLATEN	CP2 Titanium
3	1	PISTON SEAL NUT	CP2 Titanium
4	1	PRESSURE VESSEL	Aluminum 6061-T6
5	1	TOP PLATEN	CP2 Titanium
6	1	ACTUATOR CARRIER	CP2 Titanium
7	3	SPACER	Stainless Steel
8	1	PLAIN BEARING	Phosphor bronze
9	1	PISTON SEAL CARRIER	CP2 Titanium
10	1	PISTON SEAL RETAINER	Phosphor bronze
11	2	END CAP	CP2 Titanium
300	1	HYDRAULIC ACTUATOR	ENERPAC CST572
301	2	STATIC SEAL	BS 010 VITON
302	1	STATIC SEAL	BS 006 VITON
303	2	STATIC SEAL	BS 010 VITON
304	1	PISTON SEAL	BS 006 VITON
305	1	TOP PISTON	HSS DRILL BLANK 1/8X100
306	1	BOTTOM PISTON	HSS DRILL BLANK 1/8X33
307	1	TOP PISTON	316 cold drawn Stainless Steel tube 1/8x98
308	1	BOTTOM PISTON	316 cold drawn Stainless Steel tube 1/8x31
309	3	M5X90 Cylinder Head Cap Screw	Stainless Steel
310	1	M4X8 Hexagon head screws	Stainless Steel
311	3	M5x4 Hex Nut	Stainless Steel
312	3	M5 Spring Washer	Stainless Steel
313	3	M5x58 Socket Head Cap Screw	Stainless Steel
314	3	M5 belville washer	Stainless Steel
315	3	M5 Low Head Socket Cap Screw	Stainless Steel

ITEM 305 AND 306 CONFIGURATIONS

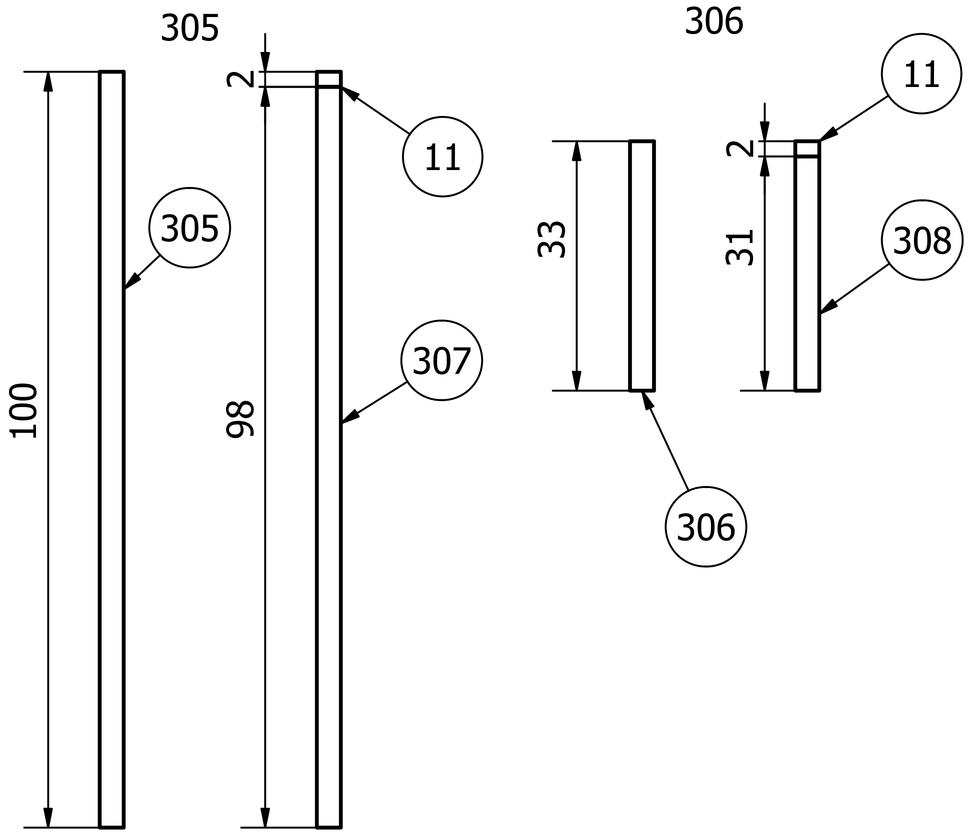

The information contained in this drawing is the sole property of The University Of Edinburgh. Any reproduction in part or whole without the written permission of The University Of Edinburgh is prohibited

REV	DESCRIPTION	NAME	DATE
01	New pressure vessel and new pistons	Dr. Butler	13/11/18

Designed by	Date	Draftsman	Date	Weight	Quantity	Format	Scale
Dr. Ian Butler	13/01/2017	I. Febbrari		0.870 kg		A2	1 : 1

Edition: 01
Sheet: 1 / 1

The University Of Edinburgh
School Of GeoSciences

Assembly name: MJÖLNIR X-RAY TRANSPARENT PRESS
Job code

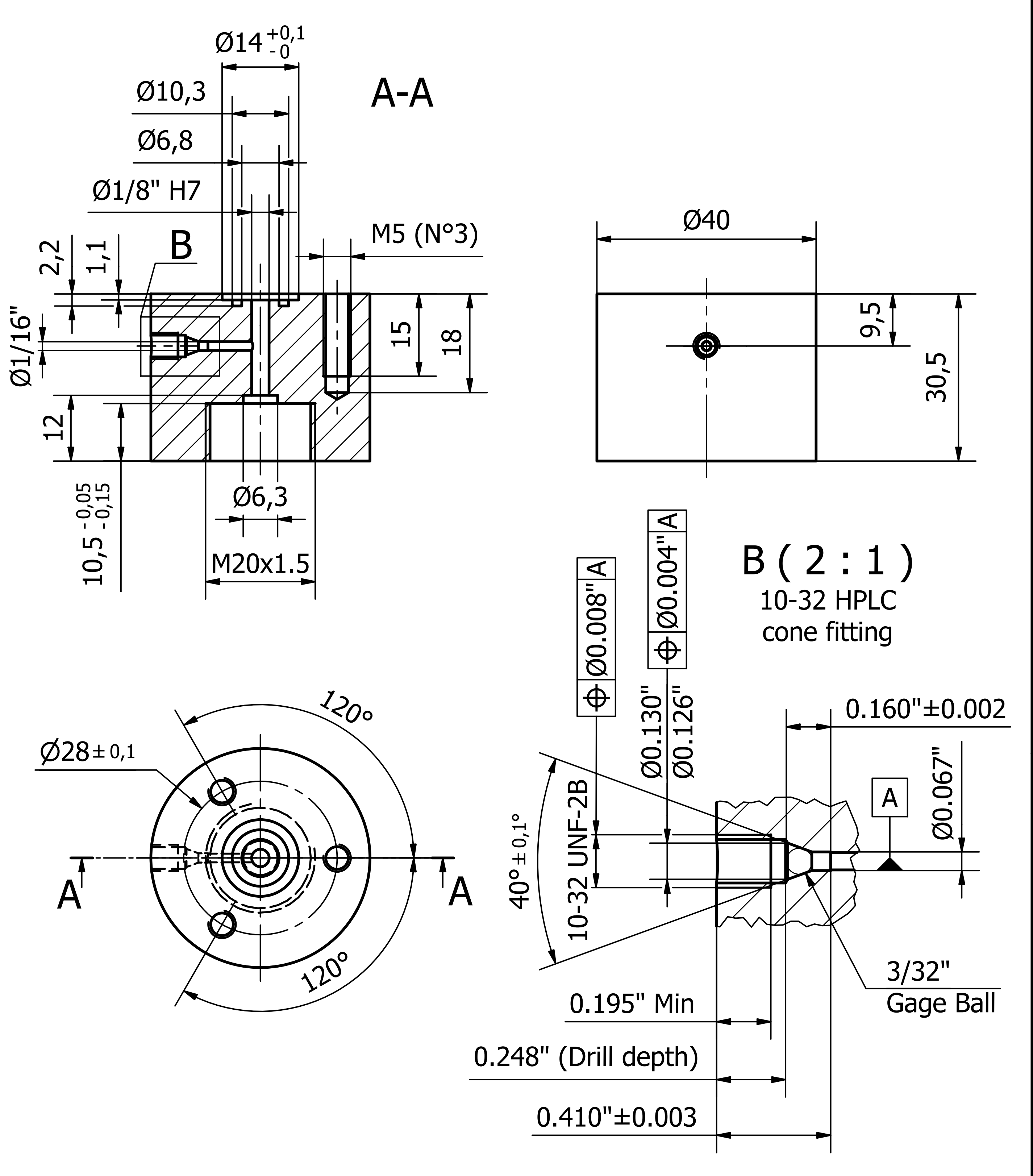

The information contained in this drawing is the sole property of The University Of Edinburgh. Any reproduction in part or whole without the written permission of The University Of Edinburgh is prohibited

REV	DESCRIPTION				NAME	DATE
Designed by: Dr. Ian Butler	Date: 16/01/2017	Draftsman: I. Febbrari	Date	Heat treatment / Surface treatment	Format: A4	Scale: 1 : 1
- ÷ XX	XX ÷ XXX	XXX ÷ XXXX	OVER XXXX	Material: CP2 Titanium	Weight: 0.155 kg	Quantity: 1
±0.1	±0.2	±0.5	±1			
**The University Of Edinburgh** School Of GeoSciences				Part name: BOTTOM PLATEN		
				Job code	Edition: 00	Sheet: 1 / 1

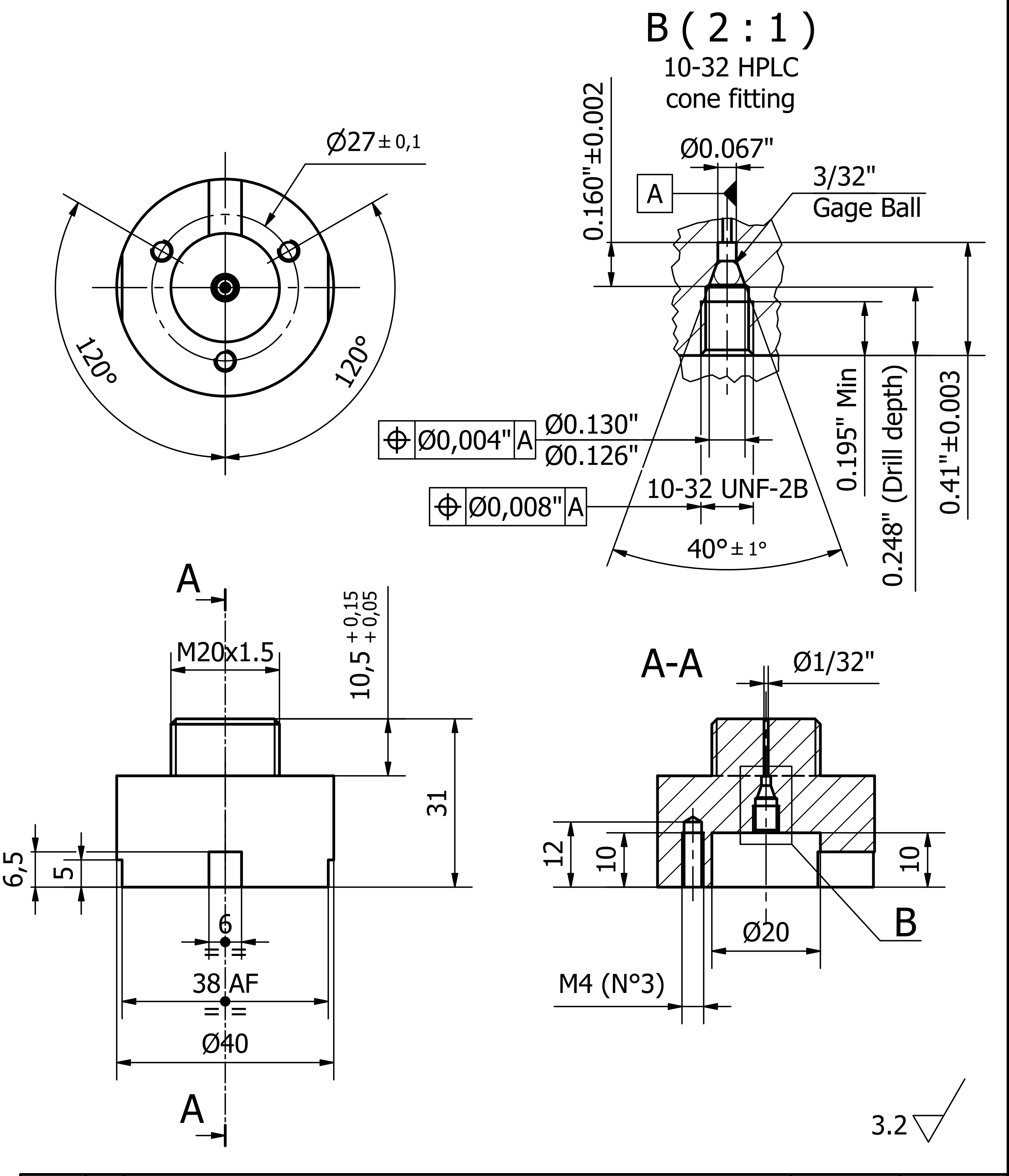

The information contained in this drawing is the sole property of The University Of Edinburgh. Any reproduction in part or whole without the written permission of The University Of Edinburgh is prohibited

REV	DESCRIPTION	NAME	DATE

Designed by	Date	Draftsman	Date	Heat treatment / Surface treatment	Format	Scale
Dr. Ian Butler	26/01/2017	I. Febbrari			A4	1 : 1

- ÷ XX	XX ÷ XXX	XXX ÷ XXXX	OVER XXXX	Material	Weight	Quantity
±0.1	±0.2	±0.5	±1	CP2 Titanium	0.113 kg	1

The University Of Edinburgh
School Of GeoSciences

Part name		
MUSHROOM		
Job code	Edition 00	Sheet 1 / 1

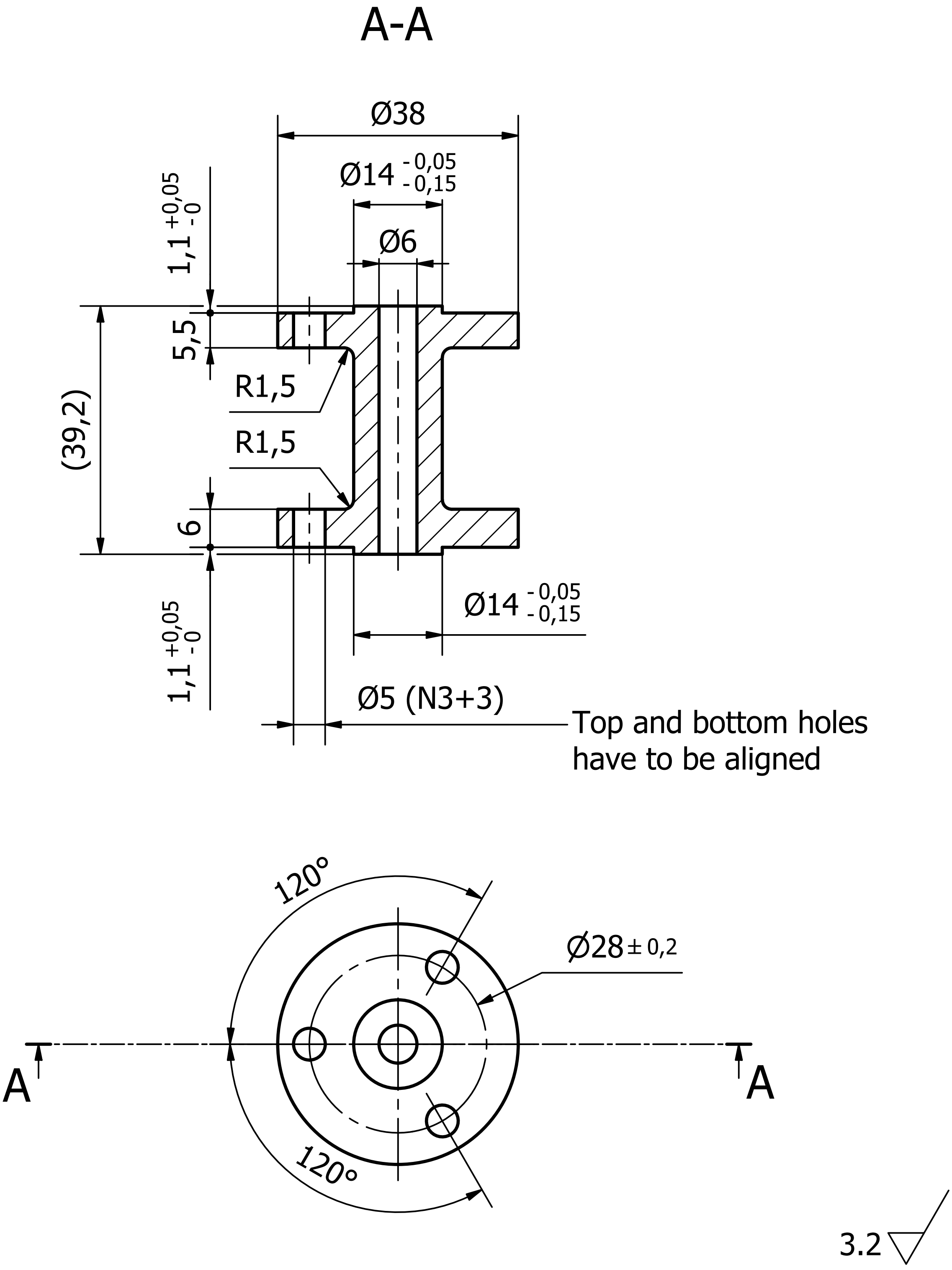

The information contained in this drawing is the sole property of The University Of Edinburgh. Any reproduction in part or whole without the written permission of The University Of Edinburgh is prohibited

REV	DESCRIPTION	NAME	DATE

Designed by	Date	Draftsman	Date	Heat treatment / Surface treatment	Format	Scale
Dr. Ian Butler	16/01/2017	I. Febbrari			A4	1 : 1

- ÷ XX	XX ÷ XXX	XXX ÷ XXXX	OVER XXXX	Material	Weight	Quantity
±0.1	±0.2	±0.5	±1	Aluminum 6061-T6	0.042 kg	1

The University Of Edinburgh
School Of GeoSciences

Part name
PRESSURE VESSEL

Job code	Edition	Sheet
	00	1 / 1

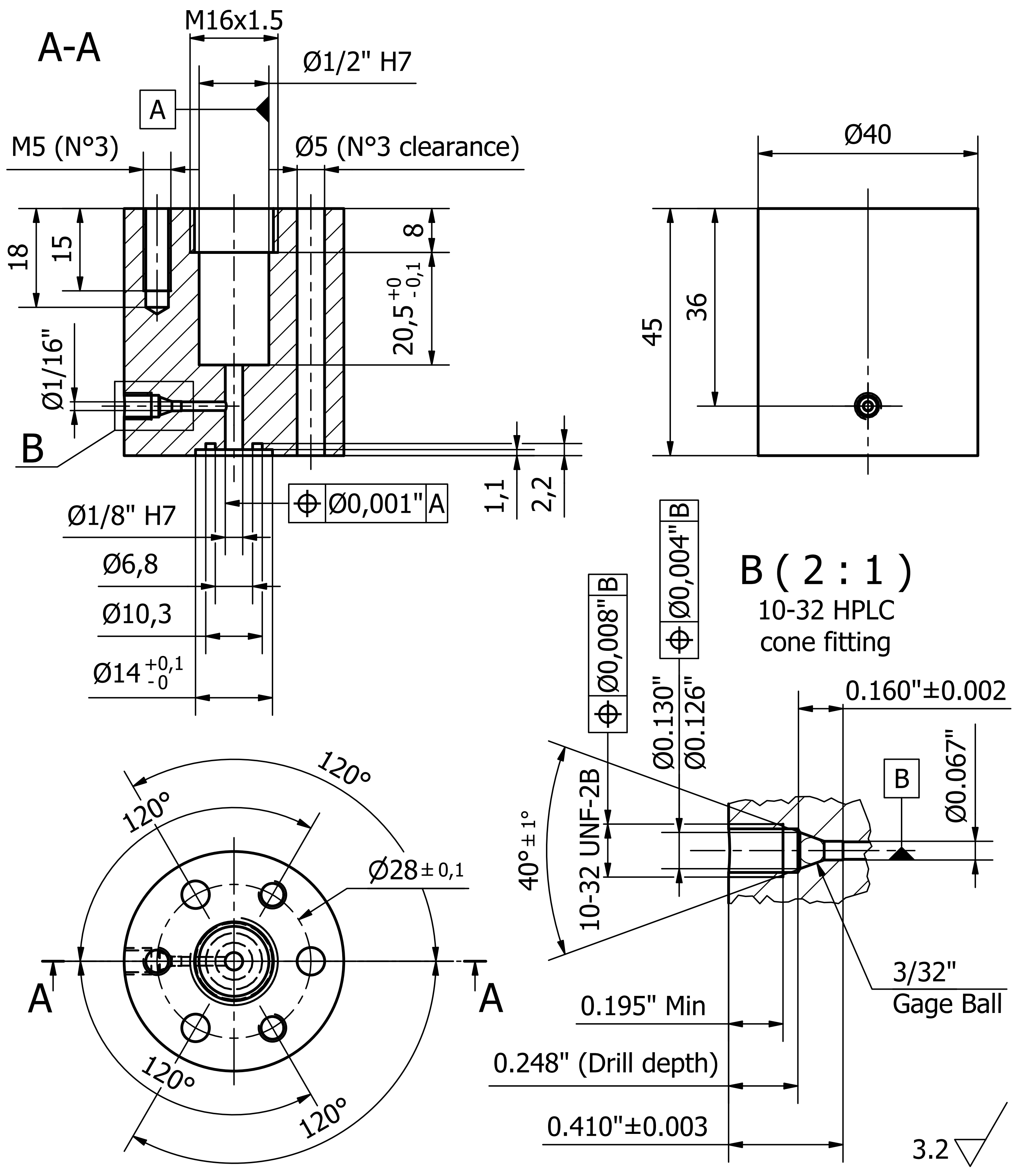

The information contained in this drawing is the sole property of The University Of Edinburgh. Any reproduction in part or whole without the written permission of The University Of Edinburgh is prohibited

REV	DESCRIPTION	NAME	DATE

Designed by	Date	Draftsman	Date	Heat treatment / Surface treatment	Format	Scale
Dr. Ian Butler	16/01/2017	I. Febbrari			A4	1 : 1

- ÷ XX	XX ÷ XXX	XXX ÷ XXXX	OVER XXXX	Material	Weight	Quantity
±0.1	±0.2	±0.5	±1	CP2 Titanium	0.220 kg	1

The University Of Edinburgh
School Of GeoSciences

Part name	Edition	Sheet
TOP PLATEN		
Job code	00	1 / 1

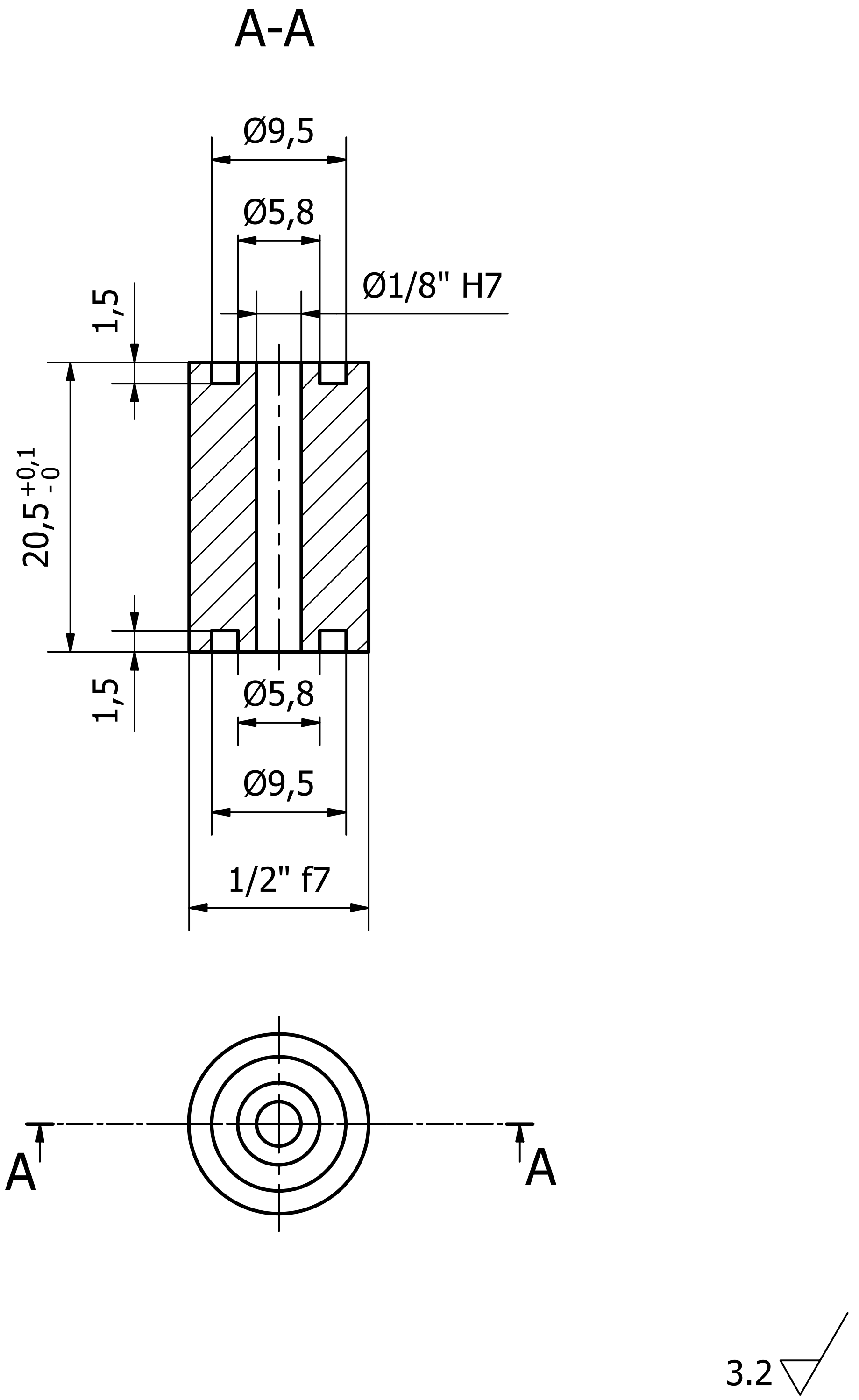

The information contained in this drawing is the sole property of The University Of Edinburgh. Any reproduction in part or whole without the written permission of The University Of Edinburgh is prohibited

REV	DESCRIPTION			NAME	DATE
Designed by: Dr. Ian Butler	Date: 27/01/2017	Supervisor/PHD Student	Date	Format: A4	Scale: 2: 1
- ÷ XX: ±0.1	XX ÷ XXX: ±0.2	XXX ÷ XXXX: ±0.5	OVER XXXX: ±1	Weight: 0.020 kg	Quantity: 1
Heat treatment / Surface treatment		Material: Phosphor Bronze			

The University Of Edinburgh
School Of GeoSciences

Part name		
PLAIN BEARING		
Job code	Edition: 00	Sheet: 1 / 1

A-A

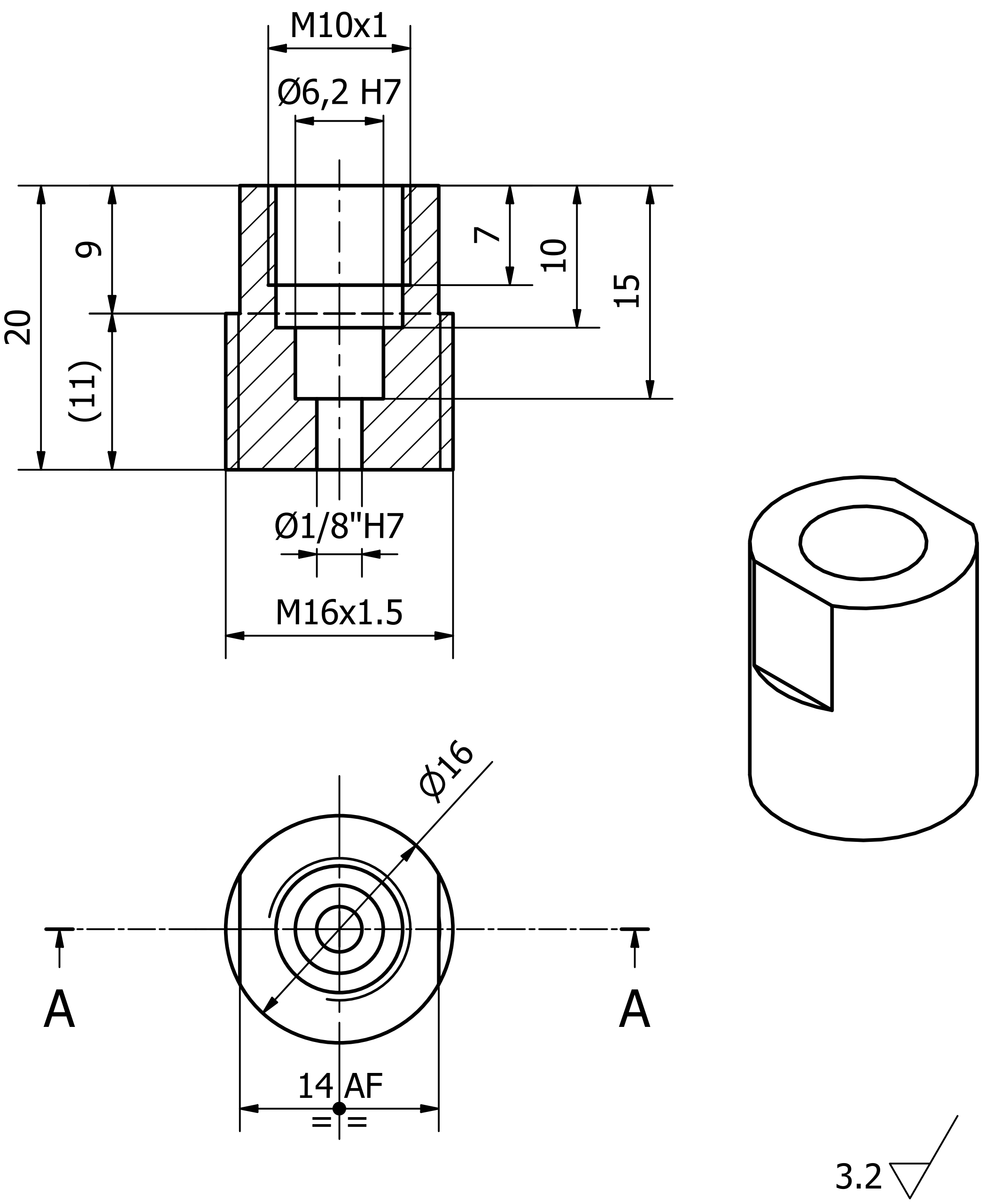

The information contained in this drawing is the sole property of The University Of Edinburgh. Any reproduction in part or whole withoutthe written permission of The University Of Edinburgh is prohibited

REV	DESCRIPTION		NAME	DATE

Designed by	Date	Draftsman	Date	Heat treatment / Surface treatment	Format	Scale
Dr. Ian Butler	22/06/2016	I. Febbrari			A4	2: 1

- ÷ XX	XX ÷ XXX	XXX ÷ XXXX	OVER XXXX	Material	Weight	Quantity
±0.1	±0.2	±0.5	±1	CP2 Titanium	0.014 kg	1

THE UNIVERSITY OF EDINBURGH

The University Of Edinburgh
School Of GeoSciences

Part name
PISTON SEAL CARRIER

Job code

Edition	Sheet
00	1 / 1

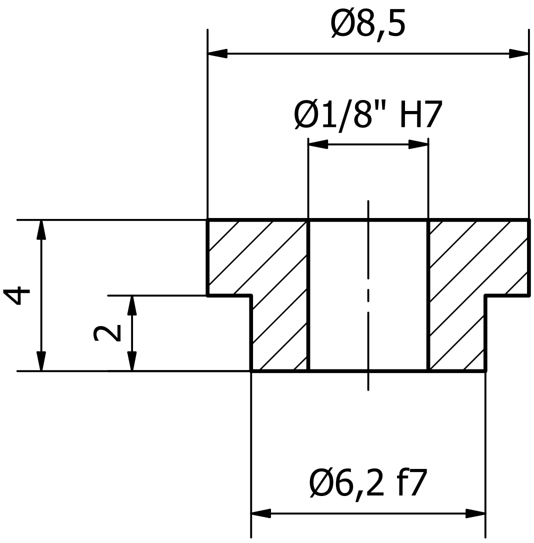

The information contained in this drawing is the sole property of The University Of Edinburgh. Any reproduction in part or whole withoutthe written permission of The University Of Edinburgh is prohibited

REV	DESCRIPTION	NAME	DATE

Designed by	Date	Draftsman	Date	Heat treatment / Surface treatment	Format	Scale
Dr. Ian Butler	22/06/2016	I. Febbrari			A4	5 : 1

- ÷ XX	XX ÷ XXX	XXX ÷ XXXX	OVER XXXX	Material	Weight	Quantity
±0.1	±0.2	±0.5	±1	Phosphor Bronze	0.001 kg	1

The University Of Edinburgh
School Of GeoSciences

Part name
PISTON SEAL RETAINER

Job code	Edition	Sheet
	00	1 / 1

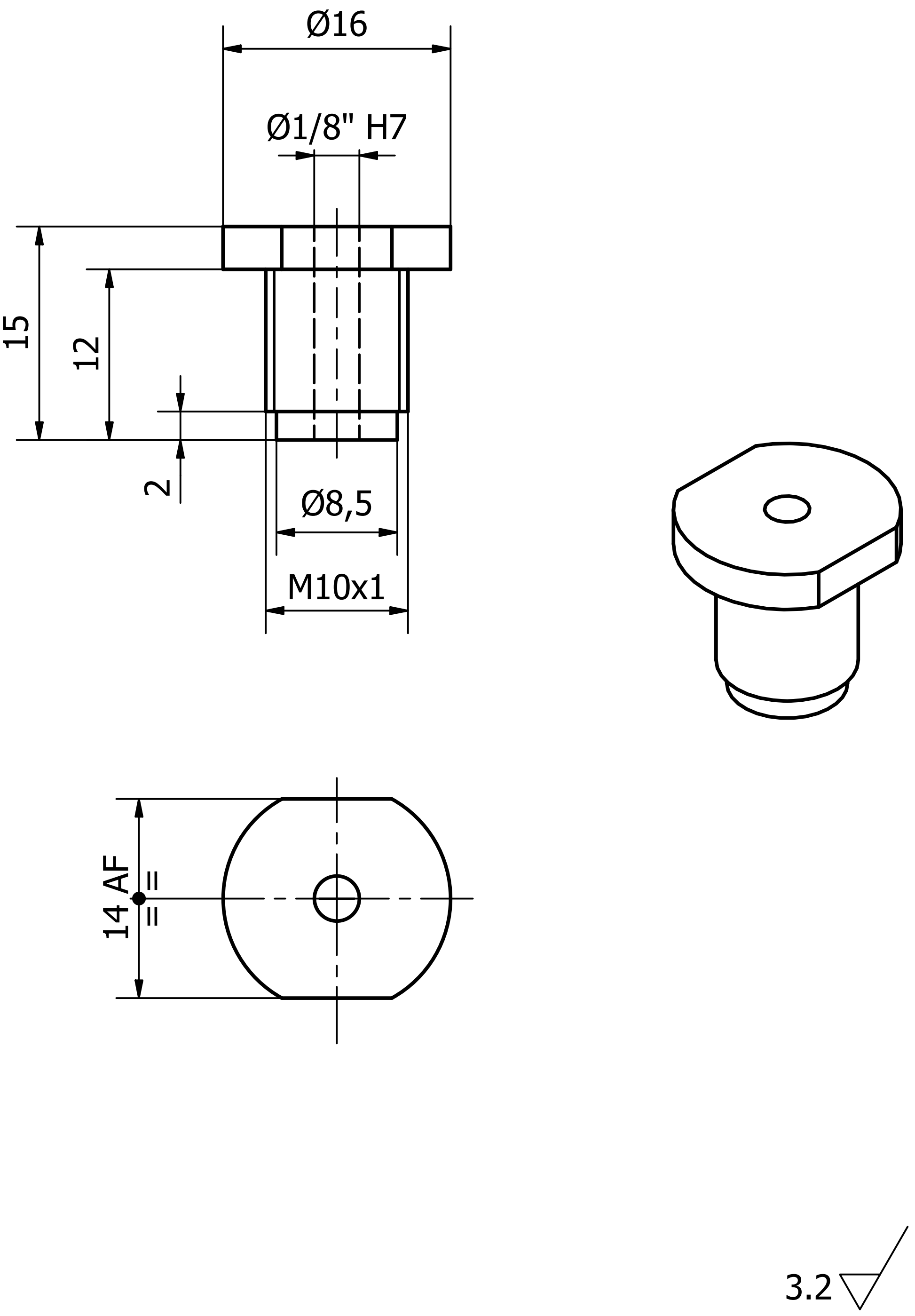

REV	DESCRIPTION	NAME	DATE

Designed by	Date	Draftsman	Date	Heat treatment / Surface treatment	Format	Scale
Dr. Ian Butler	22/06/2016	I. Febbrari			A4	2: 1

- ÷ XX	XX ÷ XXX	XXX ÷ XXXX	OVER XXXX	Material	Weight	Quantity
±0.1	±0.2	±0.5	±1	CP2 Titanium	0.006 kg	1

The University Of Edinburgh
School Of GeoSciences

Part name		
PISTON SEAL NUT		
Job code	Edition	Sheet
	00	1 / 1

The information contained in this drawing is the sole property of The University Of Edinburgh. Any reproduction in part or whole withoutthe written permission of The University Of Edinburgh is prohibited

A-A

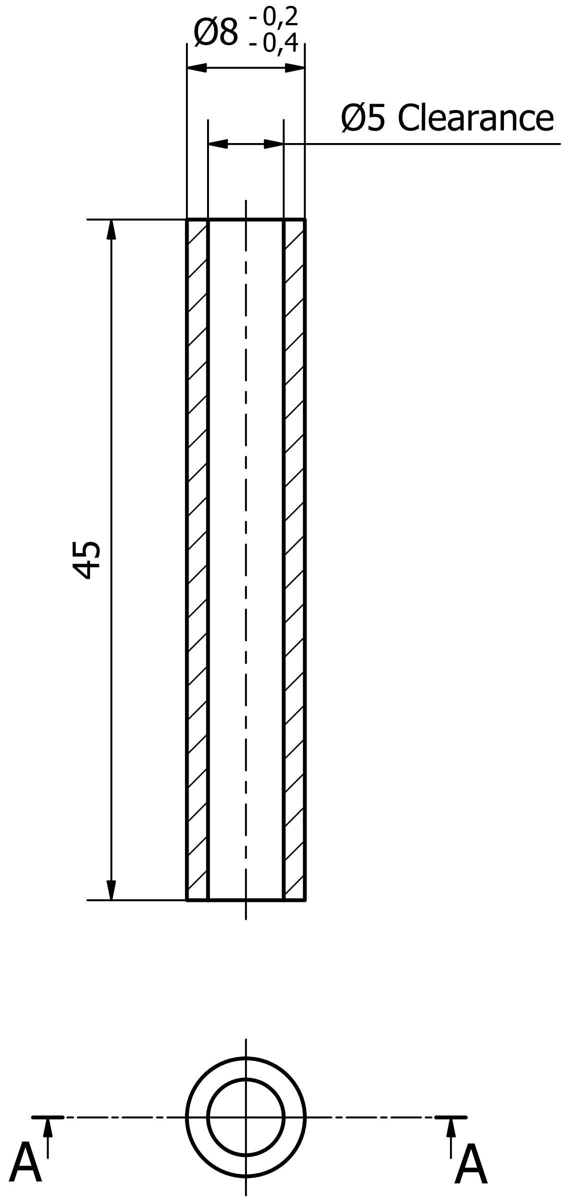

REV	DESCRIPTION	NAME	DATE

Designed by	Date	Draftsman	Date	Heat treatment / Surface treatment	Format	Scale
Dr. Ian Butler	27/01/2017	I. Febbrari			A4	2: 1

- ÷ XX	XX ÷ XXX	XXX ÷ XXXX	OVER XXXX	Material	Weight	Quantity
±0.1	±0.2	±0.5	±1	Stainless Steel	0.010 kg	3

The University Of Edinburgh
School Of GeoSciences

Part name		
SPACER		
Job code	Edition 00	Sheet 1 / 1

The information contained in this drawing is the sole property of The University Of Edinburgh. Any reproduction in part or whole without the written permission of The University Of Edinburgh is prohibited

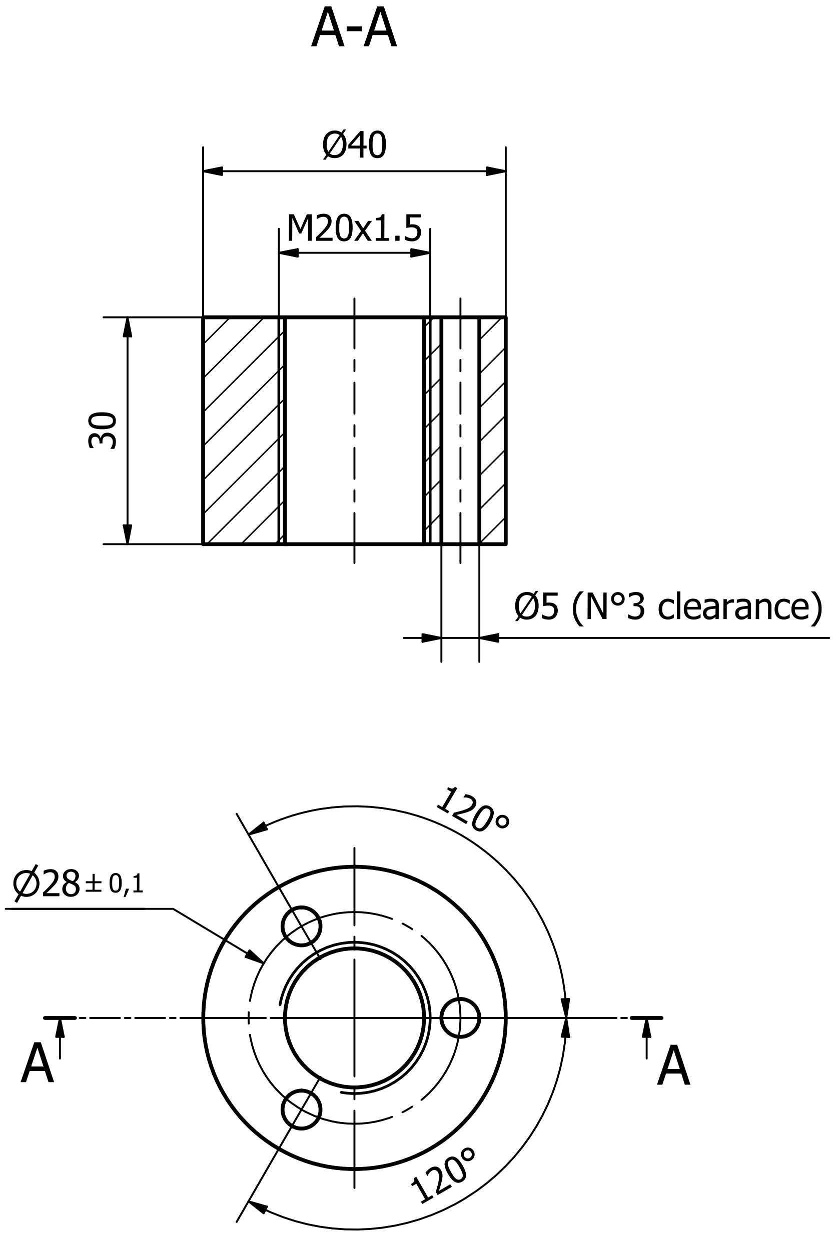

The information contained in this drawing is the sole property of The University Of Edinburgh. Any reproduction in part or whole without the written permission of The University Of Edinburgh is prohibited

REV	DESCRIPTION			NAME	DATE
Designed by: Dr. Ian Butler	Date: 16/01/2017	Draftsman: I. Febbrari	Date:	Format: A4	Scale: 1 : 1
- ÷ XX: ±0.1	XX ÷ XXX: ±0.2	XXX ÷ XXXX: ±0.5	OVER XXXX: ±1	Weight: 0.126 kg	Quantity: 1

Heat treatment / Surface treatment:

Material: CP2 Titanium

The University Of Edinburgh
School Of GeoSciences

Part name: ACTUATOR CARRIER

Job code:

Edition	Sheet
00	1 / 1

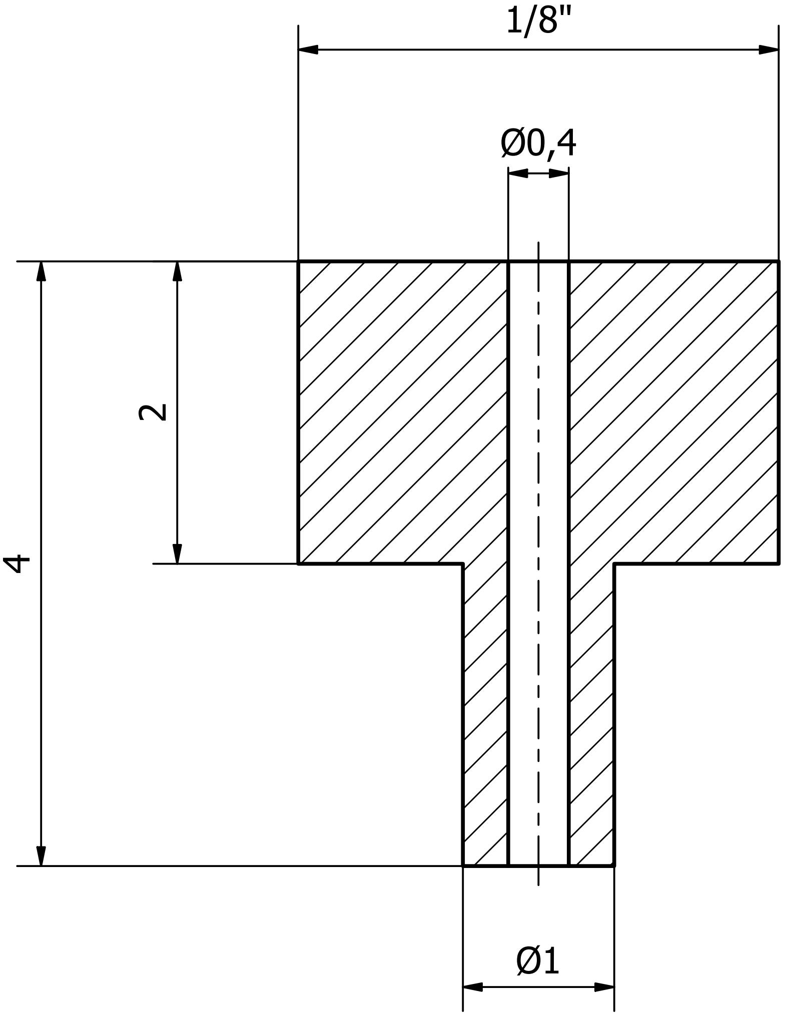

3.2

REV	DESCRIPTION	NAME	DATE

Designed by	Date	Draftsman	Date	Heat treatment / Surface treatment	Format	Scale
Dr. Ian Butler	27/01/2017	I. Febbrari			A4	20 : 1

- ÷ XX	XX ÷ XXX	XXX ÷ XXXX	OVER XXXX	Material	Weight	Quantity
±0.1	±0.2	±0.5	±1	CP2 Titanium	0.000 kg	

The University Of Edinburgh
School Of GeoSciences

Part name: END CAP

Job code	Edition	Sheet
	00	1 / 1

The information contained in this drawing is the sole property of The University Of Edinburgh. Any reproduction in part or whole without the written permission of The University Of Edinburgh is prohibited
