# Supplementary material for: Mjölnir: a miniature triaxial rock deformation apparatus for 4D synchrotron X-ray microtomography
Source: J Synchrotron Radiat. 2020 Oct 16;27(Pt 6):1681–7. doi: 10.1107/S160057752001173X (PMC7642963; doi:10.1107/S160057752001173X)
Supplement: Supplementary file 2 [file s-27-01681-sup2.pdf]

JOURNAL OF
SYNCHROTRON
RADIATION

Volume 27 (2020)

Supporting information for article:

Mjölnir: A miniature triaxial rock deformation apparatus for 4D synchrotron X-ray microtomography.

Ian Butler, Florian Fusseis, Alexis Cartwright-Taylor and Michael Flynn

S1. Technical CAD drawings for Mjölnir

Drawings provided as pdf files and Autodesk Inventor files. The pdfs are produced directly from the CAD technical drawings.

S2. Instructions for assembly of Mjolnir

Step numbers refer to the images that comprise Figure S1. Numbers in parenthesis after components refer to the bill of material (see Figure 1 and CAD drawings)

1] The first stage requires the bottom platen (2), mushroom (1), the bottom piston (either 306 or the assembly of 308 and 11) and a BS006 o-ring (302).

2] Insert the bottom piston through the bottom platen and place the BS006 o-ring over the piston end. Screw in the mushroom to compress the o ring seal.

3] Mount the bottom platen and mushroom assembly in a bench vice. Fit a BS010 o-ring (301) to the o-ring groove on the bottom platen.

4] The sample is sleeved with 25mm of 1/8” ID 3/16” OD silicone tubing. The sleeve must not touch either the bottom or top platen.

5] The sealing of the sleeve over the top and bottom pistons requires care. To aid assembly a jig can be readily prepared from M5 threaded rod sleeved with brass tube and topped by a piston guide containing a central hole which is a sliding fit for the top piston (305 or the assembly of 307 and 11).

6] To seal the sleeve two wire loops (0.35mm, 29 SWG, tinned copper wire) are twisted tight using pliers to slightly compress the silicone sleeve. Cut off excess wire and bend the short stubs of twisted wire flat against the sleeve and away from the sample to prevent the wire being captured in the images. Two loops should be used on both the top and bottom piston. Ensure that the sample remains seated between the two pistons, with no lateral movement and no gaps between the sample ends and the pistons.

7] The top platen (5) is attached to the pressure vessel (4) using M5 x 58 socket head cap screws (313), each with an M5 split spring washer (312) and an M5 x 4 hex nut (311). M5 x 58 cap screws were made by cutting down M5 x 60 cap screws. The cap screws should be tightened evenly (torque driver at 2 Nm) to fully compress the spring washers. Inspect the interface between the top platen and pressure vessel flange to ensure that the pressure vessel is not tilted relative to the top platen. Two BS010 o-rings (303) are fitted to the plain bearing (8), which is inserted into the top platen.

8] Remove the piston guide plate. Hold the piston-sample assembly steady. Remove the guide plate support legs and lower the top platen-pressure vessel assembly over the top piston. Secure the lower

flange of the pressure vessel using 3 M5 x 15 socket head cap screws (315), each with a Bellville washer (314). The screw head profile should be 4mm or less. Tighten the screws evenly and inspect the joint between the bottom platen and the pressure vessel flange to ensure that they are not tilted with respect to each other.

9] Place the piston seal carrier (9) over the top piston and tighten. Push a BS006 (304) o-ring over the top piston, followed by the piston seal retainer (10) and the piston seal nut (3). Tighten the piston seal nut to the limit of its travel and then loosen fractionally to ensure free axial movement of the top piston.

10] The final stage requires m5 x 90 cylinder head high tensile steel cap screws (309), spacer tubes (7), the actuator carrier (6) and the Enerpac CST572 actuator. The threaded tip of the actuator piston can be fitted with an M4 x 5 hex bolt to facilitate loading of the top piston. Some M5 x 90 cap screws may require modification to prevent interference with the actuator cylinder thread.

11] Locate the actuator carrier onto the top platen with the M5 x 90 cylinder head screws tightened evenly. Screw the actuator into the actuator carrier.

12] Tighten the actuator gently against the top piston to prevent the sleeve being pushed in between the sample and pistons on addition of confining pressure. Beware, it is easy to break weak samples at this stage by over tightening. Mount cell on the rotary stage, and check if the sample is accurately placed between the pistons using x-ray imaging. If the sample needs centring, the actuator can be unscrewed fractionally and the sample centred by applying 2-3 bar of confining pressure.

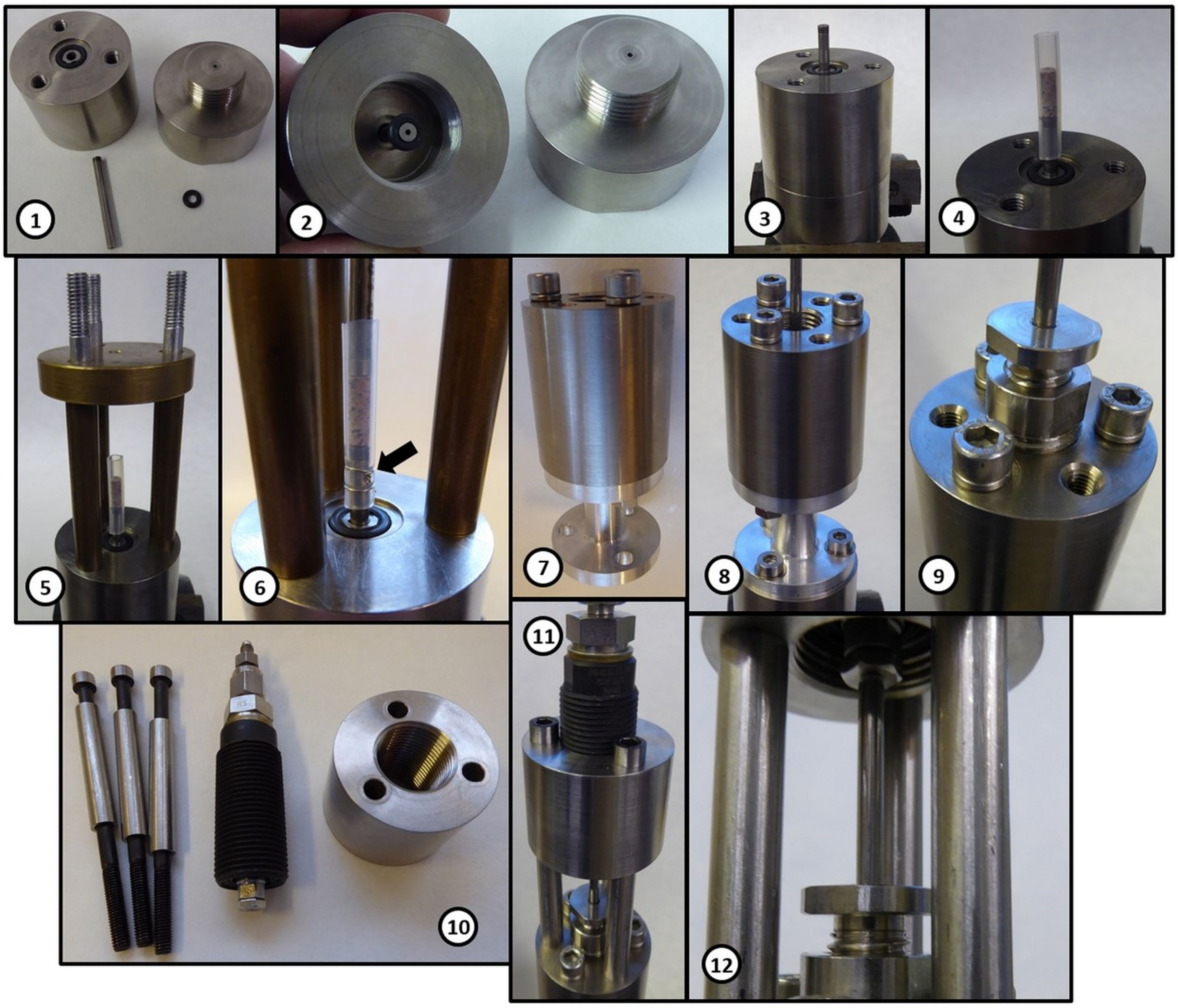

Figure S1 Assembly sequence for Mjölnir. Each step is explained in detail in supporting information S2.
